# 7-Chloro-4-[(*E*)-2-(2-methoxy­benzyl­idene)hydrazin-1-yl]quinoline monohydrate

**DOI:** 10.1107/S1600536810006586

**Published:** 2010-02-27

**Authors:** Marcus V. N. de Souza, R. Alan Howie, Edward R. T. Tiekink, James L. Wardell, Solange M. S. V. Wardell, Carlos R. Kaiser

**Affiliations:** a Instituto de Tecnologia em Farmacos, Fundação Oswaldo Cruz (FIOCRUZ), FarManguinhos, Rua Sizenando Nabuco, 100, Manguinhos, 21041-250 Rio de Janeiro, RJ, Brazil; b Department of Chemistry, University of Aberdeen, Old Aberdeen AB15 5NY, Scotland; c Department of Chemistry, University of Malaya, 50603 Kuala Lumpur, Malaysia; d Centro de Desenvolvimento Tecnológico em Saúde (CDTS), Fundação Oswaldo Cruz (FIOCRUZ), Casa Amarela, Campus de Manguinhos, Av. Brasil 4365, 21040-900 Rio de Janeiro, RJ, Brazil; e CHEMSOL, 1 Harcourt Road, Aberdeen AB15 5NY, Scotland; f Departamento de Química Orgânica, Instituto de Química, Universidade Federal do Rio de Janeiro, 21945-970 Rio de Janeiro, RJ, Brazil

## Abstract

In the title hydrate, C_17_H_14_ClN_3_O·H_2_O, the dihedral angle between the quinoline fused-ring system and the benzene ring is 13.4 (2)° and the conformation about the C=N bond is *E*. In the crystal, N_h_—H⋯O_w_ and O_w_—H⋯N_q_ (h = hydro­zone, w = water and q = quinoline) hydrogen bonds generate a two-dimenstional network in the *ac* plane. A weak C—H⋯O inter­action helps to consolidate the packing.

## Related literature

For background to the pharmacological activity of quinoline derivatives, see: Warshakoon *et al.* (2006 ▶). For recent studies into quinoline-based anti-malarials, see: Andrade *et al.* (2007 ▶); de Souza *et al.* (2005 ▶). For related structures, see: Kaiser *et al.* (2009 ▶); de Souza *et al.* (2009 ▶, 2010 ▶). For the structure of the isomeric 2-meth­oxy structure, see: de Lima Ferreira *et al.* (2010 ▶).
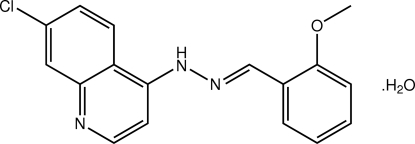


## Experimental

### 

#### Crystal data

C_17_H_14_ClN_3_O·H_2_O
*M* *_r_* = 329.78
Monoclinic, 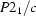

*a* = 3.9202 (2) Å
*b* = 24.5084 (17) Å
*c* = 16.1212 (11) Å
β = 91.639 (4)°
*V* = 1548.26 (17) Å^3^
*Z* = 4
Mo *K*α radiation
μ = 0.26 mm^−1^
*T* = 120 K
0.62 × 0.03 × 0.02 mm

#### Data collection

Nonius KappaCCD diffractometer
Absorption correction: multi-scan (*SADABS*; Sheldrick, 2007 ▶) *T*_min_ = 0.735, *T*_max_ = 0.995
11507 measured reflections
2716 independent reflections
1769 reflections with *I* > 2σ(*I*)
*R*_int_ = 0.096

#### Refinement

*R*[*F* ^2^ > 2σ(*F* ^2^)] = 0.093
*wR*(*F* ^2^) = 0.260
*S* = 1.04
2716 reflections
215 parameters
H atoms treated by a mixture of independent and constrained refinement
Δρ_max_ = 0.40 e Å^−3^
Δρ_min_ = −0.45 e Å^−3^

### 

Data collection: *COLLECT* (Hooft, 1998 ▶); cell refinement: *DENZO* (Otwinowski & Minor, 1997 ▶) and *COLLECT*; data reduction: *DENZO* and *COLLECT*; program(s) used to solve structure: *SHELXS97* (Sheldrick, 2008 ▶); program(s) used to refine structure: *SHELXL97* (Sheldrick, 2008 ▶); molecular graphics: *ORTEP-3* (Farrugia, 1997 ▶) and *DIAMOND* (Brandenburg, 2006 ▶); software used to prepare material for publication: *publCIF* (Westrip, 2010 ▶).

## Supplementary Material

Crystal structure: contains datablocks global, I. DOI: 10.1107/S1600536810006586/hb5340sup1.cif

Structure factors: contains datablocks I. DOI: 10.1107/S1600536810006586/hb5340Isup2.hkl

Additional supplementary materials:  crystallographic information; 3D view; checkCIF report

## Figures and Tables

**Table 1:** Hydrogen-bond geometry (Å, °)

*D*—H⋯*A*	*D*—H	H⋯*A*	*D*⋯*A*	*D*—H⋯*A*
N2—H2*N*⋯O1*W*	0.88	2.08	2.928 (7)	161
O1*W*—H1*W*⋯N1^i^	0.81 (9)	2.30 (9)	3.030 (8)	150 (8)
O1*W*—H2*W*⋯N1^ii^	0.82 (9)	2.03 (9)	2.820 (7)	163 (8)
C5—H5⋯O1*W*	0.95	2.43	3.358 (8)	166

Symmetry codes: (i) 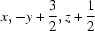; (ii) 
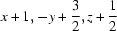
.

**Fig. 1.**
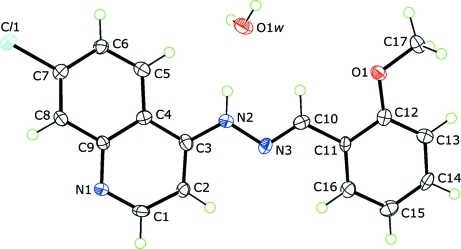
Molecular structures of the asymmetric unit in (I) showing displacement ellipsoids at the 50% probability level.

**Fig. 2.**
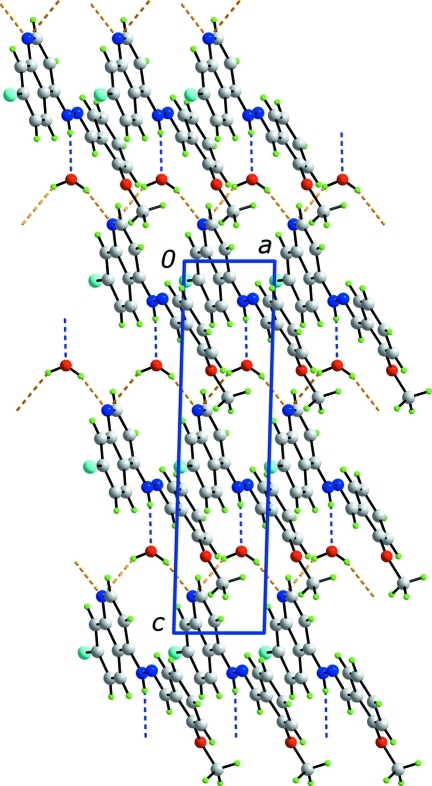
View of the 2-D supramolecular array in the ac plane of (I) showing the O–H···N and N–H···O hydrogen bonding as orange and blue dashed lines, respectively. Colour code: Cl, cyan; O, red; N, blue; C, grey; and H, green.

**Fig. 3.**
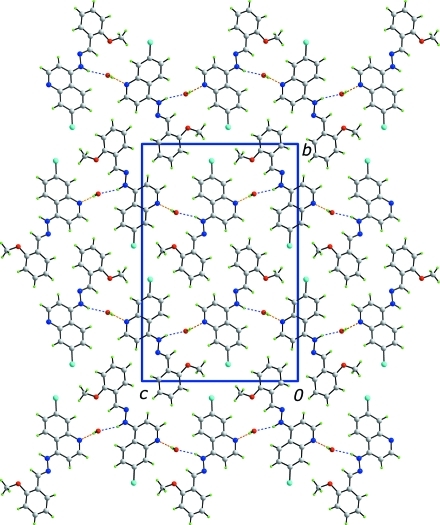
A view of the stacking of layers in (I). The O–H···N and N–H···O hydrogen bonding as orange and blue dashed lines, respectively. Colour code: Cl, cyan; O, red; N, blue; C, grey; and H, green.

## supplementary crystallographic information

### Comment

Quinoline derivatives are known to display pharmacological potential (Warshakoon *et al.*, 2006) and are being investigated for their anti-malarial activity (Andrade *et al.* 2007; de Souza *et al.*, 2005). Structural studies on quinoline derivatives augment the biological investigations (Kaiser *et al.*, 2009; de Souza *et al.*, 2009; de Souza *et al.*, 2010; de Lima Ferreira *et al.*, 2010) and as a part of these studies, the crystal structure of the title hydrate, (I), was investigated.

The most significant twist in the quinoline molecule of (I), Fig. 1, occurs around the C10–C11 bond as seen in the N3–C10–C11–C16 torsion angle of 6.9 (9) °. This accounts for the dihedral angle of 13.4 (2) ° formed between the quinoline fused-ring system and the benzene ring. The conformation about the C10═N3 bond [1.282 (8) Å] is *E*. The crystal packing is stabilised by a variety of hydrogen bonding interactions, Table 1. The water molecule accepts a hydrogen bond from the hydrazone-N2 atom and bridges two symmetry related molecules by forming donor interactions with quinoline-N1 atoms; the water-O atom also participates in a C–H···O contact, Table 1. The result of the hydrogen bonding is the formation of a 2-D supramolecular array in the *ac* plane, Fig. 2, and these stack along the *b* axis, Fig. 3.

### Experimental

A solution of 7-chloro-4-quinolinylhydrazine(0.2 g, 1.03 mmol) and 2-methoxybenzaldehyde (1.2 mmol) in EtOH (5 ml) was maintained at room temperature overnight and rotary evaporated. The solid residue, was washed with cold Et_2_O (3 x 10 ml) and recrystallised from EtOH; m.pt. 459-461 K, yield 82%. The sample for the X-ray study was slowly grown from moist EtOH and was found to be the monohydrate. MS/ESI: [M—H]: 310.8. IR νmax (cm-1; KBr disc): 3190 (N—H), 1578 (C=N).

### Refinement

The N- and C-bound H atoms were geometrically placed (N–H = 0.88 Å and C–H = 0.95–0.98 Å) and refined as riding with *U_iso_*(H) = 1.2–1.5*U_eq_*(C,*N*). The water-bound H atoms were located from a difference map and refined with *U_iso_*(H) = 1.5*U_eq_*(O).

### Crystal data

**Table:**

C_17_H_14_ClN_3_O·H_2_O	*F*(000) = 688
*M**_r_* = 329.78	*D*_x_ = 1.415 Mg m^−^^3^
Monoclinic, *P*2_1_/*c*	Mo *K*α radiation, λ = 0.71073 Å
Hall symbol: -P 2ybc	Cell parameters from 9260 reflections
*a* = 3.9202 (2) Å	θ = 2.9–27.5°
*b* = 24.5084 (17) Å	µ = 0.26 mm^−^^1^
*c* = 16.1212 (11) Å	*T* = 120 K
β = 91.639 (4)°	Needle, colourless
*V* = 1548.26 (17) Å^3^	0.62 × 0.03 × 0.02 mm
*Z* = 4	

### Data collection

**Table:**

Nonius KappaCCD diffractometer	2716 independent reflections
Radiation source: Enraf Nonius FR591 rotating anode	1769 reflections with *I* > 2σ(*I*)
10 cm confocal mirrors	*R*_int_ = 0.096
Detector resolution: 9.091 pixels mm^-1^	θ_max_ = 25.0°, θ_min_ = 3.0°
φ and ω scans	*h* = −4→4
Absorption correction: multi-scan (*SADABS*; Sheldrick, 2007)	*k* = −29→29
*T*_min_ = 0.735, *T*_max_ = 0.995	*l* = −19→19
11507 measured reflections	

### Refinement

**Table:**

Refinement on *F*^2^	Primary atom site location: structure-invariant direct methods
Least-squares matrix: full	Secondary atom site location: difference Fourier map
*R*[*F*^2^ > 2σ(*F*^2^)] = 0.093	Hydrogen site location: inferred from neighbouring sites
*wR*(*F*^2^) = 0.260	H atoms treated by a mixture of independent and constrained refinement
*S* = 1.04	*w* = 1/[σ^2^(*F*_o_^2^) + (0.1*P*)^2^ + 10.4045*P*] where *P* = (*F*_o_^2^ + 2*F*_c_^2^)/3
2716 reflections	(Δ/σ)_max_ = 0.001
215 parameters	Δρ_max_ = 0.40 e Å^−^^3^
0 restraints	Δρ_min_ = −0.45 e Å^−^^3^

### Special details

**Table:**

Geometry. All s.u.'s (except the s.u. in the dihedral angle between two l.s. planes) are estimated using the full covariance matrix. The cell s.u.'s are taken into account individually in the estimation of s.u.'s in distances, angles and torsion angles; correlations between s.u.'s in cell parameters are only used when they are defined by crystal symmetry. An approximate (isotropic) treatment of cell s.u.'s is used for estimating s.u.'s involving l.s. planes.
Refinement. Refinement of *F*^2^ against ALL reflections. The weighted *R*-factor wR and goodness of fit *S* are based on *F*^2^, conventional *R*-factors *R* are based on *F*, with *F* set to zero for negative *F*^2^. The threshold expression of *F*^2^ > 2σ(*F*^2^) is used only for calculating *R*-factors(gt) etc. and is not relevant to the choice of reflections for refinement. *R*-factors based on *F*^2^ are statistically about twice as large as those based on *F*, and *R*- factors based on ALL data will be even larger.

### Fractional atomic coordinates and isotropic or equivalent isotropic displacement parameters (Å^2^)

**Table:**

	*x*	*y*	*z*	*U*_iso_*/*U*_eq_	
Cl1	0.0208 (4)	0.57108 (6)	0.05313 (10)	0.0256 (5)	
O1	1.3298 (11)	0.93299 (18)	0.2940 (3)	0.0232 (10)	
N1	0.1966 (13)	0.7497 (2)	−0.0985 (3)	0.0201 (12)	
N2	0.6619 (13)	0.8261 (2)	0.1080 (3)	0.0214 (12)	
H2N	0.6968	0.8080	0.1547	0.026*	
N3	0.7680 (12)	0.8795 (2)	0.1015 (3)	0.0195 (12)	
C1	0.2920 (16)	0.8018 (3)	−0.0989 (4)	0.0232 (15)	
H1	0.2504	0.8220	−0.1484	0.028*	
C2	0.4479 (15)	0.8288 (2)	−0.0322 (4)	0.0183 (13)	
H2	0.5157	0.8658	−0.0377	0.022*	
C3	0.5046 (15)	0.8016 (2)	0.0426 (4)	0.0203 (14)	
C4	0.3926 (15)	0.7459 (2)	0.0470 (4)	0.0176 (13)	
C5	0.4234 (15)	0.7141 (3)	0.1204 (4)	0.0223 (14)	
H5	0.5240	0.7296	0.1692	0.027*	
C6	0.3094 (16)	0.6611 (2)	0.1217 (4)	0.0206 (14)	
H6	0.3293	0.6402	0.1712	0.025*	
C7	0.1640 (15)	0.6384 (2)	0.0496 (4)	0.0176 (13)	
C8	0.1307 (16)	0.6672 (3)	−0.0225 (4)	0.0213 (14)	
H8	0.0329	0.6504	−0.0707	0.026*	
C9	0.2419 (15)	0.7222 (2)	−0.0257 (4)	0.0180 (13)	
C10	0.9303 (15)	0.8980 (3)	0.1657 (4)	0.0211 (14)	
H10	0.9774	0.8745	0.2115	0.025*	
C11	1.0442 (14)	0.9551 (2)	0.1693 (4)	0.0164 (13)	
C12	1.2380 (14)	0.9726 (2)	0.2387 (4)	0.0181 (14)	
C13	1.3295 (16)	1.0271 (3)	0.2466 (4)	0.0237 (15)	
H13	1.4596	1.0391	0.2938	0.028*	
C14	1.2299 (16)	1.0641 (3)	0.1851 (4)	0.0222 (14)	
H14	1.2908	1.1014	0.1909	0.027*	
C15	1.0422 (16)	1.0471 (3)	0.1152 (4)	0.0249 (15)	
H15	0.9778	1.0725	0.0731	0.030*	
C16	0.9495 (15)	0.9922 (2)	0.1078 (4)	0.0212 (14)	
H16	0.8211	0.9802	0.0604	0.025*	
C17	1.5235 (16)	0.9495 (3)	0.3666 (4)	0.0242 (15)	
H17A	1.7439	0.9642	0.3501	0.036*	
H17B	1.5613	0.9179	0.4030	0.036*	
H17C	1.3976	0.9777	0.3962	0.036*	
O1W	0.7223 (14)	0.7893 (2)	0.2807 (3)	0.0304 (12)	
H1W	0.53 (2)	0.785 (3)	0.300 (5)	0.046*	
H2W	0.89 (2)	0.780 (3)	0.309 (5)	0.046*	

### Atomic displacement parameters (Å^2^)

**Table:**

	*U*^11^	*U*^22^	*U*^33^	*U*^12^	*U*^13^	*U*^23^
Cl1	0.0319 (9)	0.0186 (8)	0.0263 (9)	−0.0044 (7)	−0.0002 (7)	0.0044 (7)
O1	0.028 (2)	0.023 (2)	0.018 (2)	0.0005 (19)	−0.0047 (18)	−0.001 (2)
N1	0.027 (3)	0.019 (3)	0.015 (3)	−0.002 (2)	−0.003 (2)	0.002 (2)
N2	0.027 (3)	0.020 (3)	0.017 (3)	−0.002 (2)	−0.003 (2)	0.002 (2)
N3	0.022 (3)	0.015 (3)	0.022 (3)	−0.002 (2)	0.004 (2)	−0.002 (2)
C1	0.026 (3)	0.026 (4)	0.017 (4)	0.000 (3)	−0.005 (3)	0.008 (3)
C2	0.023 (3)	0.015 (3)	0.017 (3)	−0.001 (2)	0.004 (3)	−0.004 (3)
C3	0.018 (3)	0.020 (3)	0.022 (4)	0.002 (3)	−0.003 (3)	−0.004 (3)
C4	0.016 (3)	0.020 (3)	0.017 (3)	0.001 (2)	0.005 (2)	0.001 (3)
C5	0.023 (3)	0.025 (3)	0.019 (4)	0.002 (3)	−0.002 (3)	0.000 (3)
C6	0.029 (3)	0.015 (3)	0.018 (4)	0.002 (3)	0.001 (3)	−0.001 (3)
C7	0.019 (3)	0.017 (3)	0.018 (3)	0.002 (2)	0.008 (2)	−0.002 (3)
C8	0.023 (3)	0.024 (3)	0.017 (4)	−0.004 (3)	−0.004 (3)	−0.001 (3)
C9	0.023 (3)	0.016 (3)	0.015 (3)	0.000 (2)	−0.002 (3)	−0.002 (3)
C10	0.019 (3)	0.021 (3)	0.023 (4)	0.001 (3)	0.002 (3)	0.005 (3)
C11	0.014 (3)	0.016 (3)	0.018 (3)	−0.004 (2)	0.001 (2)	−0.003 (3)
C12	0.014 (3)	0.019 (3)	0.022 (4)	0.001 (2)	0.006 (2)	−0.004 (3)
C13	0.027 (3)	0.026 (3)	0.018 (4)	−0.001 (3)	0.000 (3)	0.001 (3)
C14	0.026 (3)	0.015 (3)	0.026 (4)	−0.007 (3)	0.011 (3)	−0.003 (3)
C15	0.027 (3)	0.019 (3)	0.029 (4)	0.006 (3)	0.003 (3)	0.003 (3)
C16	0.024 (3)	0.018 (3)	0.021 (4)	0.001 (3)	0.004 (3)	−0.004 (3)
C17	0.022 (3)	0.030 (4)	0.021 (4)	−0.002 (3)	−0.002 (3)	−0.005 (3)
O1W	0.028 (3)	0.039 (3)	0.024 (3)	0.000 (2)	−0.005 (2)	0.004 (2)

### Geometric parameters (Å, °)

**Table:**

Cl1—C7	1.744 (6)	C7—C8	1.362 (9)
O1—C12	1.360 (7)	C8—C9	1.419 (9)
O1—C17	1.434 (7)	C8—H8	0.9500
N1—C1	1.330 (8)	C10—C11	1.471 (8)
N1—C9	1.361 (8)	C10—H10	0.9500
N2—C3	1.348 (8)	C11—C16	1.388 (9)
N2—N3	1.379 (7)	C11—C12	1.401 (8)
N2—H2N	0.8800	C12—C13	1.388 (9)
N3—C10	1.282 (8)	C13—C14	1.391 (9)
C1—C2	1.389 (9)	C13—H13	0.9500
C1—H1	0.9500	C14—C15	1.391 (9)
C2—C3	1.391 (9)	C14—H14	0.9500
C2—H2	0.9500	C15—C16	1.399 (9)
C3—C4	1.436 (8)	C15—H15	0.9500
C4—C9	1.420 (8)	C16—H16	0.9500
C4—C5	1.420 (9)	C17—H17A	0.9800
C5—C6	1.372 (9)	C17—H17B	0.9800
C5—H5	0.9500	C17—H17C	0.9800
C6—C7	1.396 (9)	O1W—H1W	0.81 (9)
C6—H6	0.9500	O1W—H2W	0.82 (9)
			
C12—O1—C17	117.2 (5)	N1—C9—C4	123.4 (5)
C1—N1—C9	116.6 (5)	C8—C9—C4	118.6 (6)
C3—N2—N3	119.7 (5)	N3—C10—C11	120.7 (6)
C3—N2—H2N	120.2	N3—C10—H10	119.6
N3—N2—H2N	120.2	C11—C10—H10	119.6
C10—N3—N2	114.6 (5)	C16—C11—C12	119.9 (6)
N1—C1—C2	124.9 (6)	C16—C11—C10	121.4 (5)
N1—C1—H1	117.6	C12—C11—C10	118.7 (5)
C2—C1—H1	117.6	O1—C12—C13	124.3 (6)
C1—C2—C3	119.9 (6)	O1—C12—C11	115.7 (5)
C1—C2—H2	120.1	C13—C12—C11	120.0 (6)
C3—C2—H2	120.1	C12—C13—C14	119.6 (6)
N2—C3—C2	121.6 (6)	C12—C13—H13	120.2
N2—C3—C4	121.2 (6)	C14—C13—H13	120.2
C2—C3—C4	117.2 (5)	C15—C14—C13	120.9 (6)
C9—C4—C5	119.1 (5)	C15—C14—H14	119.5
C9—C4—C3	117.9 (5)	C13—C14—H14	119.5
C5—C4—C3	122.9 (6)	C14—C15—C16	119.1 (6)
C6—C5—C4	120.8 (6)	C14—C15—H15	120.4
C6—C5—H5	119.6	C16—C15—H15	120.4
C4—C5—H5	119.6	C11—C16—C15	120.3 (6)
C5—C6—C7	119.3 (6)	C11—C16—H16	119.8
C5—C6—H6	120.3	C15—C16—H16	119.8
C7—C6—H6	120.3	O1—C17—H17A	109.5
C8—C7—C6	122.0 (6)	O1—C17—H17B	109.5
C8—C7—Cl1	119.7 (5)	H17A—C17—H17B	109.5
C6—C7—Cl1	118.3 (5)	O1—C17—H17C	109.5
C7—C8—C9	120.1 (6)	H17A—C17—H17C	109.5
C7—C8—H8	119.9	H17B—C17—H17C	109.5
C9—C8—H8	119.9	H1W—O1W—H2W	118 (9)
N1—C9—C8	118.0 (5)		
			
C3—N2—N3—C10	−176.6 (6)	C7—C8—C9—C4	1.1 (9)
C9—N1—C1—C2	3.4 (9)	C5—C4—C9—N1	178.9 (6)
N1—C1—C2—C3	−2.1 (10)	C3—C4—C9—N1	−0.4 (9)
N3—N2—C3—C2	0.5 (9)	C5—C4—C9—C8	−0.8 (9)
N3—N2—C3—C4	179.6 (5)	C3—C4—C9—C8	179.9 (6)
C1—C2—C3—N2	178.5 (6)	N2—N3—C10—C11	−177.0 (5)
C1—C2—C3—C4	−0.6 (9)	N3—C10—C11—C16	6.9 (9)
N2—C3—C4—C9	−177.4 (6)	N3—C10—C11—C12	−176.3 (6)
C2—C3—C4—C9	1.7 (8)	C17—O1—C12—C13	2.3 (9)
N2—C3—C4—C5	3.3 (9)	C17—O1—C12—C11	−178.9 (5)
C2—C3—C4—C5	−177.6 (6)	C16—C11—C12—O1	−177.5 (5)
C9—C4—C5—C6	0.0 (9)	C10—C11—C12—O1	5.6 (8)
C3—C4—C5—C6	179.3 (6)	C16—C11—C12—C13	1.3 (9)
C4—C5—C6—C7	0.5 (9)	C10—C11—C12—C13	−175.6 (6)
C5—C6—C7—C8	−0.1 (9)	O1—C12—C13—C14	178.2 (6)
C5—C6—C7—Cl1	−179.9 (5)	C11—C12—C13—C14	−0.5 (9)
C6—C7—C8—C9	−0.7 (10)	C12—C13—C14—C15	−0.6 (10)
Cl1—C7—C8—C9	179.1 (5)	C13—C14—C15—C16	0.9 (10)
C1—N1—C9—C8	177.7 (6)	C12—C11—C16—C15	−1.0 (9)
C1—N1—C9—C4	−2.0 (9)	C10—C11—C16—C15	175.8 (6)
C7—C8—C9—N1	−178.6 (6)	C14—C15—C16—C11	−0.1 (10)

### Hydrogen-bond geometry (Å, °)

**Table:**

*D*—H···*A*	*D*—H	H···*A*	*D*···*A*	*D*—H···*A*
N2—H2N···O1W	0.88	2.08	2.928 (7)	161
O1W—H1W···N1^i^	0.81 (9)	2.30 (9)	3.030 (8)	150 (8)
O1W—H2W···N1^ii^	0.82 (9)	2.03 (9)	2.820 (7)	163 (8)
C5—H5···O1W	0.95	2.43	3.358 (8)	166

Symmetry codes: (i) *x*, −*y*+3/2, *z*+1/2; (ii) *x*+1, −*y*+3/2, *z*+1/2.
